# Depression and Depressive Symptoms in Glaucoma: A Systematic Review and Meta‐Analysis of Comparative, Prevalence, and Longitudinal Evidence

**DOI:** 10.1155/da/7344513

**Published:** 2026-08-14

**Authors:** Xuan Liao, Chenxuan Liu, Mengxiong Luo, Weihua Yang

**Affiliations:** ^1^ Department of Ophthalmology, Affiliated Hospital, North Sichuan Medical College, Nanchong, Sichuan, China, nsmc.edu.cn; ^2^ Shenzhen Eye Hospital, Shenzhen Eye Medical Center, Southern Medical University, Shenzhen, Guangdong, China, fimmu.com

**Keywords:** depression, depressive symptoms, glaucoma, meta-analysis, observational studies, systematic review, visual impairment

## Abstract

**Background:**

Evidence suggests that people with glaucoma may experience more depressive symptoms than those without glaucoma, but prior estimates have been limited by heterogeneous ascertainment and insufficient separation of comparative, prevalence, and longitudinal evidence.

**Methods:**

We searched PubMed, Embase, Cochrane Library, Web of Science, SinoMed, Wanfang, VIP, CNKI, ChiCTR, and ClinicalTrials.gov to March 27, 2026. Eligible studies reported depression outcomes among individuals with glaucoma; studies included in the primary comparative analysis additionally required a non‐glaucoma comparison group. Two reviewers independently screened studies, extracted data, and assessed quality using the Newcastle–Ottawa Scale (NOS) for cohort/case‐control studies and Joanna Briggs Institute (JBI) tools for cross‐sectional studies. Random‐effects meta‐analyses used restricted maximum likelihood (REML) as the primary estimator; DerSimonian‐Laird models were sensitivity analyses. Certainty of evidence was assessed using the GRADE framework adapted for observational evidence.

**Results:**

Forty‐nine studies were included qualitatively. Sixteen studies including 14,030 glaucoma participants and 4,472,723 controls contributed to the primary odds ratio meta‐analysis. Glaucoma was associated with higher odds of depression (OR, 2.80; 95% CI, 1.66–4.74; *I* ^2^ = 95.95%; approximate prediction interval 0.32–24.47). Results remained significant across sensitivity analyses excluding self‐reported glaucoma studies, unclear glaucoma ascertainment, and a very large database study. Thirty‐eight studies including 21,705 patients with glaucoma contributed to the single‐group prevalence meta‐analysis; pooled depression prevalence was 26.03% (95% CI, 21.10%–31.64%; prediction interval 5.98%–66.04%). Longitudinal evidence was limited and heterogeneous; one UK Biobank cohort suggested a longitudinal association with incident hospitalized depression among participants with glaucoma (HR, 1.54; 95% CI, 1.01–2.34). GRADE certainty was very low for the comparative, prevalence, and longitudinal evidence domains.

**Conclusions:**

Very low‐certainty observational evidence suggests that depression or depressive symptoms are more common among individuals with glaucoma; however, whether glaucoma precedes, contributes to, or results from depressive symptoms remains uncertain. The observed association may reflect complex bidirectional relationships between glaucoma‐related visual burden and depressive symptoms. Ophthalmic care should incorporate pragmatic depression awareness and referral pathways, while future longitudinal studies should use standardized glaucoma and depression ascertainment with adequate adjustment for visual, clinical, and socioeconomic confounders.

## 1. Introduction

Glaucoma is a chronic optic neuropathy and a leading cause of irreversible visual impairment worldwide [1–5]. Its long disease course, need for continuous monitoring and treatment, uncertainty about progression, and potential effects on visual function can place a sustained psychological burden on patients [2–4, 6–8]. Depression is common in chronic illness and may present with persistent low mood, anhedonia, fatigue, sleep or appetite disturbance, impaired concentration, guilt or hopelessness, and functional decline [9–11]. In glaucoma, these symptoms may interact with visual disability, treatment burden, and reduced health‐related quality of life [6–8].

Previous primary studies have examined depression in glaucoma through comparative case‐control or cross‐sectional analyses [12–16], single‐arm prevalence estimates [17–20], and longitudinal datasets [21–23]. Important uncertainty remains because the evidence differs widely in study design, clinical setting, glaucoma subtype and ascertainment, depression definition, and adjustment for visual function, comorbidity, and treatment‐related factors. To address these issues, we constructed an analysis‐specific extraction dataset and separately synthesized comparative association, single‐group prevalence, and longitudinal evidence.

The objective of this systematic review and meta‐analysis was to quantify the association between glaucoma and depression compared with non‐glaucoma controls, estimate the prevalence of depression among glaucoma patients, and summarize longitudinal evidence narratively where effect measures could not be pooled.

## 2. Methods

### 2.1. Data Sources and Search Strategy

See Supporting Information 1: PRISMA 2020 checklist. We searched PubMed, Embase, Cochrane Library, Web of Science, SinoMed, Wanfang Database, VIP Database, CNKI, ChiCTR, and ClinicalTrials.gov from inception to March 27, 2026. Search terms combined controlled vocabulary and free‐text terms for depression or depressive symptoms with glaucoma, primary open‐angle glaucoma, and primary angle‐closure glaucoma. See Supporting Information 2: Search strategies and results [24].

Two reviewers independently screened titles and abstracts, retrieved potentially eligible full texts, and assessed eligibility. Disagreements were resolved by discussion and, when needed, consultation with a senior reviewer. The review protocol was prospectively registered in PROSPERO (CRD420261352502).

### 2.2. Inclusion and Exclusion Criteria

We included observational studies, including cross‐sectional, case‐control, nested case‐control, and cohort studies, that reported depression, depressive symptoms, or incident depression among participants with glaucoma. For the primary odds ratio meta‐analysis, studies were required to provide extractable 2 × 2 data comparing glaucoma with a non‐glaucoma control group. For the single‐group prevalence meta‐analysis, studies were eligible if they reported the number of patients with glaucoma and depression or sufficient data to derive this number.

Conference abstracts were eligible only when sufficient extractable data were available; incomplete reports were flagged during quality assessment and sensitivity analyses.

Glaucoma could be diagnosed by clinical examination, medical records, diagnostic codes, standardized ophthalmic criteria, or self‐report; the ascertainment method was coded for subgroup and sensitivity analyses. Depression could be assessed by clinical diagnosis, diagnostic codes, structured interview, or validated screening scales such as PHQ‐9 and HADS‐D [25, 26], as well as CES‐D, GDS, BDI‐II, SDS/Zung, HAM‐D/HDRS, or related instruments. We excluded laboratory‐only studies, reviews, editorials, case reports, studies without depression‐specific data, studies in which glaucoma data could not be separated from other ocular diseases, and reports without extractable denominators or effect estimates.

### 2.3. Data Extraction

Two reviewers extracted study identifiers, country or region, study design and setting, sample size, glaucoma subtype (including POAG, PACG, secondary glaucoma, pseudoexfoliative glaucoma, ocular hypertension, and glaucoma suspect status when reported), glaucoma ascertainment method, depression ascertainment method and threshold, events and denominators, reported adjusted estimates where available, visual‐function or vision‐related quality‐of‐life indicators when reported, including NEI VFQ‐derived measures [27], treatment‐related variables, and notes relevant to sensitivity analyses. Subtype‐specific effect estimates were extracted when available; however, quantitative pooling was performed only when sufficient comparable data were available. See Supporting Information 3: Data extraction file.

Coder training consisted of a pilot extraction round on a subset of included studies, reconciliation of definitions for glaucoma ascertainment, depression thresholds, and eligible comparator groups, and the development of a shared coding dictionary. Discrepancies were logged and adjudicated by consensus. Formal interrater agreement was not calculated, which is acknowledged as a limitation.

### 2.4. Quality Assessment

Cohort and case‐control studies were evaluated using the Newcastle–Ottawa Scale (NOS) [28]. Cross‐sectional studies were evaluated using the Joanna Briggs Institute (JBI) critical appraisal checklist for analytical cross‐sectional studies [29]. The quality assessment was performed independently by two reviewers. Studies were not excluded solely by quality score, but quality concerns were incorporated into sensitivity analyses and interpretation. Detailed item‐level quality assessments are provided in Supporting Information 4: Supporting Table S2.

We assessed the certainty of evidence for the main comparative odds‐ratio analysis, the single‐arm prevalence analysis, and the longitudinal evidence using the GRADE framework adapted for observational evidence [30]. Observational evidence was initially rated as low‐certainty and was downgraded for risk of bias, inconsistency, indirectness, imprecision, and suspected publication bias where applicable. No upgrading was applied because the estimates were derived from heterogeneous observational evidence with plausible residual confounding. Certainty judgments and domain‐specific rationales are provided in Supporting Information 4: Supporting Tables S3 (GRADE Summary) and Supporting Information 4: Table S4 (Domain Rationale).

### 2.5. Outcomes

The primary outcome was the association between glaucoma and depression, expressed as odds ratios comparing depression among glaucoma participants with non‐glaucoma controls. Odds ratios were selected as the primary association measure because most eligible studies provided case‐control frequency data or reported OR‐based estimates, whereas risk ratios were not consistently available. Given the low absolute frequency of depression events in several included datasets, ORs were retained as the common effect measure for consistency across studies.

The secondary outcome was the pooled prevalence of depression or depressive symptoms among patients with glaucoma.

Longitudinal evidence was summarized narratively because eligible studies differed in comparison groups and effect measures, and only one study provided a directly extractable glaucoma‐versus‐control hazard ratio with a 95% CI.

### 2.6. Data Synthesis and Statistical Analysis

For comparative studies, crude odds ratios and log odds ratios were calculated from extracted 2 × 2 data. Because adjusted estimates were not consistently reported and differed substantially in covariate adjustment, the primary comparative meta‐analysis used crude 2 × 2 data. Adjusted estimates were considered unsuitable for quantitative pooling because the covariate sets differed substantially across studies. Pooling adjusted estimates with nonoverlapping covariate structures may introduce additional heterogeneity and reduce interpretability. Extractable adjusted estimates were summarized narratively and tabulated in the data extraction file when available. The primary model was a random‐effects meta‐analysis fitted using restricted maximum likelihood (REML). DerSimonian‐Laird random‐effects models were used as sensitivity analyses [31]. Heterogeneity was described using the *χ* ^2^ test, *I* ^2^, *τ* ^2^, Cochran *Q*, and prediction intervals [32, 33].

For single‐group prevalence, proportions were pooled using a random‐effects metaprop approach with logit transformation and REML estimation. Exact binomial confidence intervals were calculated for study‐level prevalence estimates. Heterogeneity and prediction intervals were reported.

Subgroup analyses examined the glaucoma ascertainment method, study setting, geographical region, and depression ascertainment. Sensitivity analyses excluded self‐reported glaucoma studies, studies with unclear glaucoma confirmation, a very large electronic health record study, and extreme prevalence estimates. Because depression instruments and cut‐offs were inconsistently reported and were not directly comparable across scales, a scale‐specific threshold sensitivity analysis was not feasible; therefore, extreme‐prevalence exclusion was retained only as a pragmatic stability analysis and not as a threshold‐specific sensitivity analysis. Leave‐one‐out analyses assessed the influence. Publication bias in the main OR meta‐analysis was examined using funnel plots, Egger regression, Begg rank correlation, and trim‐and‐fill [34–36]. Comparative OR meta‐analyses were conducted in StataNow version 19.5 (StataCorp LLC, College Station, TX, USA), and single‐group prevalence analyses were conducted in R version 4.5.1 using the meta package. All tests were two‐sided, and *p* values <0.05 were considered statistically significant unless otherwise specified.

## 3. Results

### 3.1. Study Selection

**Figure 1:**
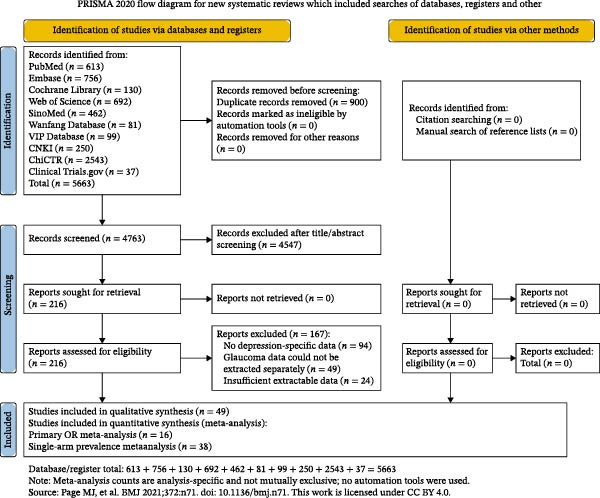
PRISMA 2020 flow diagram of study selection.

Database and registry searches identified 5663 records: PubMed (*n* = 613), Embase (*n* = 756), Cochrane Library (*n* = 130), Web of Science (*n* = 692), SinoMed (*n* = 462), Wanfang (*n* = 81), VIP (*n* = 99), CNKI (*n* = 250), ChiCTR (*n* = 2543), and ClinicalTrials.gov (*n* = 37). After removing 900 duplicates, 4763 records were screened. A total of 4547 records were excluded after title/abstract screening, leaving 216 reports for full‐text eligibility assessment. Of these, 167 were excluded because depression‐specific data were unavailable (*n* = 94), glaucoma data could not be extracted separately (*n* = 49), or extractable data were insufficient (*n* = 24). Forty‐nine studies were included in the qualitative synthesis; 16 contributed to the primary OR meta‐analysis and 38 to the single‐arm prevalence meta‐analysis (Figure 1).

### 3.2. Study Characteristics and Quality Assessment

The 49 included records represented studies from China, the United States, South Korea, Japan, Germany, India, Australia, Turkey, Brazil, Nigeria, Serbia, Canada, the United Kingdom, Israel, Taiwan, Singapore, Croatia, Mexico, and Hungary. Study designs included hospital‐ or clinic‐based cross‐sectional studies, community‐ and population‐based cross‐sectional studies, case‐control studies, retrospective electronic health record studies, and longitudinal cohort or nested case‐control analyses. Glaucoma ascertainment ranged from detailed ophthalmic examination and medical records to self‐report or administrative diagnostic codes. Depression ascertainment included screening scales, structured interviews, and diagnostic code definitions. The 49 included studies are summarized in Table 1 and Supporting Information 4: Supporting Table S2. To make citation mapping explicit, the comparative OR studies were [12–18, 21, 37–44], the single‐arm prevalence studies were [7, 8, 12–20, 37–41, 43–64], the longitudinal or supportive narrative studies were [21–23, 65], and records retained for qualitative or sensitivity‐only synthesis were [6, 66–70]. Detailed study‐level characteristics and quality assessment results are provided in Supporting Information 4: Supporting Table S2.

**Table 1:** Characteristics of included studies.

Domain	Category	Count or summary	Notes
Included evidence	Qualitative synthesis	49	All eligible records included in narrative/study‐characteristics synthesis
Included evidence	Primary comparative OR meta‐analysis	16	Studies with extractable glaucoma‐versus‐non‐glaucoma 2 × 2 data
Included evidence	Single‐arm prevalence meta‐analysis	38	Studies with extractable depression events and glaucoma denominators
Included evidence	Longitudinal narrative synthesis	4	Studies reporting incident depression or longitudinal risk‐factor evidence
Geographical region	Africa	2	Study count
Geographical region	Asia	26	Study count
Geographical region	Europe	7	Study count
Geographical region	North America	10	Study count
Geographical region	Oceania	2	Study count
Geographical region	South America	2	Study count
Study setting/design context	Other or unclear setting	13	Categorized from study design and recruitment source
Study setting/design context	Population, community, database, or registry setting	12	Categorized from study design and recruitment source
Study setting/design context	Hospital/clinic or clinical setting	24	Categorized from study design and recruitment source
Glaucoma ascertainment	Clinical, medical‐record, diagnostic‐code, or examination‐based ascertainment	43	Used for subgroup/sensitivity interpretation
Glaucoma ascertainment	Self‐reported glaucoma	5	Used for subgroup/sensitivity interpretation
Glaucoma ascertainment	Unclear or incompletely reported confirmation	1	Used for subgroup/sensitivity interpretation
Depression ascertainment	Questionnaire‐ or screening‐scale‐based assessment	44	Screening scales included PHQ, HADS, CES‐D, GDS, BDI, SDS/Zung, and HAM‐D/HDRS
Depression ascertainment	Diagnostic code, clinical diagnosis, or structured interview	5	Included diagnostic‐code definitions, clinical diagnoses, or structured‐interview diagnoses; details are provided in Supporting Information 4: Supporting Table S2
Quality assessment	NOS range for cohort/case‐control studies	4–8	Sampaio 2019 was rated as limited/not fully assessable because only an ARVO Annual Meeting abstract was available
Quality assessment	JBI range for cross‐sectional studies	4–7	Detailed study‐level and item‐level quality information is provided in Supporting Information 4: Supporting Table S2

*Note:* Analysis categories are not mutually exclusive; some studies contributed to more than one evidence category.

Abbreviations: JBI, Joanna Briggs Institute; NOS, Newcastle–Ottawa Scale; OR, odds ratio.

### 3.3. Primary OR Meta‐Analysis

**Figure 2:**
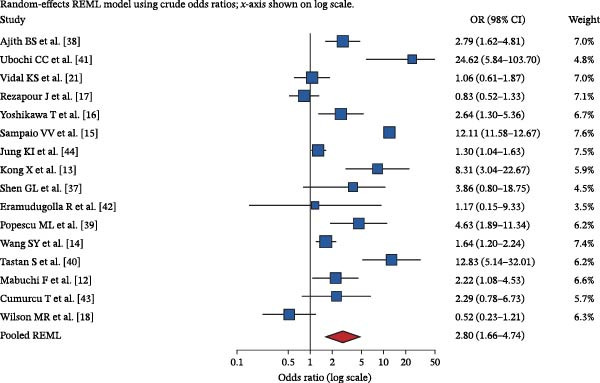
Forest plot of the association between glaucoma and depression. Random‐effects REML model; effect estimates are odds ratios. Square size is proportional to random‐effects weight. Diamond indicates pooled REML estimate; vertical line indicates OR = 1.

Sixteen comparative studies provided extractable data for the primary analysis, including 14,030 participants with glaucoma and 4,472,723 controls. Depression was reported in 2967 glaucoma participants and 104,717 controls. The REML random‐effects model showed that glaucoma was associated with higher odds of depression (OR, 2.80; 95% CI, 1.66–4.74; *z* = 3.85; *p* = 0.0001). Heterogeneity was substantial (*I* ^2^ = 95.95%; *τ* ^2^ = 0.95; *Q*(15) = 799.59; *p* < 0.001), and the approximate 95% prediction interval was wide (0.32–24.47), indicating that the magnitude and direction of association may vary across future settings. Study‐specific effect estimates are shown in Figure 2, and summary meta‐analytic findings are shown in Table 2.

**Table 2:** Main meta‐analytic findings.

Analysis	Studies	Effect estimate	95% CI	*I* ^2^ (%)	*p*‐Value	Prediction interval
Main OR meta‐analysis, REML model	16	OR, 2.80	1.66–4.74	95.95	0.0001	0.32–24.47
DL model sensitivity analysis	16	OR, 2.85	1.36–6.01	98.12	0.0058	Not applicable
Excluding self‐reported glaucoma studies	12	OR, 3.78	2.06–6.92	95.51	<0.0001	Not applicable
Excluding Yoshikawa et al. [16]	15	OR, 2.82	1.61–4.96	96.38	0.0003	Not applicable
Excluding Sampaio et al. [15]	15	OR, 2.46	1.49–4.06	89.71	0.0004	Not applicable
Clinical/medical ascertainment only	11	OR, 3.93	2.02–7.62	96.08	0.0001	Not applicable
Single‐arm prevalence meta‐analysis	38	26.03%	21.10%–31.64%	95.95	<0.0001	5.98%–66.04%
Prevalence analysis excluding extreme prevalence studies	29	25.69%	21.99%–29.78%	93.91	<0.0001	10.47%–50.54%

*Note:* Not applicable indicates that a prediction interval was not calculated for that sensitivity analysis.

Abbreviations: CI, confidence interval; DL, DerSimonian‐Laird; *I* ^2^, inconsistency statistic; OR, odds ratio; REML, restricted maximum likelihood.

### 3.4. Sensitivity and Subgroup Analyses for the Main OR

Results were robust in direction across sensitivity analyses. The DerSimonian‐Laird model produced a similar pooled estimate (OR, 2.85; 95% CI, 1.36–6.01; *I* ^2^ = 98.12%). Excluding four self‐reported glaucoma studies strengthened the association (OR, 3.78; 95% CI, 2.06–6.92). Excluding Yoshikawa 2019, in which glaucoma ascertainment was based on fundus photograph‐defined glaucomatous optic disc without visual field confirmation, yielded an OR of 2.82 (95% CI, 1.61–4.96). Excluding the very large Sampaio 2019 database study reduced heterogeneity and retained significance (OR, 2.46; 95% CI, 1.49–4.06; *I* ^2^ = 89.71%). Restriction to 11 studies with clinical, medical record, or examination‐based glaucoma ascertainment yielded an OR of 3.93 (95% CI, 2.02–7.62). Leave‐one‐out analyses ranged from OR 2.46 to 3.12, and all estimates remained statistically significant.

Subgroup analyses suggested that glaucoma ascertainment contributed to the heterogeneity. Studies using clinical or medical ascertainment showed a stronger association (OR, 3.93; 95% CI, 2.02–7.62) than self‐reported glaucoma studies (OR, 1.18; 95% CI, 0.78–1.77; P for subgroup difference = 0.002). Hospital/clinic‐based studies showed an OR of 4.10 (95% CI, 2.06–8.18), whereas population/database‐based studies showed an OR of 1.93 (95% CI, 0.90–4.12; subgroup *p* = 0.148). After correction of country/region classification, Asian studies showed an OR of 3.19 (95% CI, 1.85–5.50) and non‐Asian studies an OR of 2.39 (95% CI, 0.94–6.11; subgroup *p* = 0.601).

### 3.5. Publication Bias

See Supporting Information 5: Figure S1: Main odds‐ratio funnel plot. Egger regression did not indicate statistically significant small‐study effects (*p* = 0.555), whereas Begg rank correlation was statistically significant (*p* = 0.013). See Supporting Information 6: Figure S2: Trim‐and‐fill funnel plot. Given the very high heterogeneity and substantial differences in study design, these findings should be interpreted cautiously as possible small‐study or design‐related asymmetry rather than definitive evidence of publication bias.

### 3.6. Single‐Arm Prevalence Meta‐Analysis

**Figure 3:**
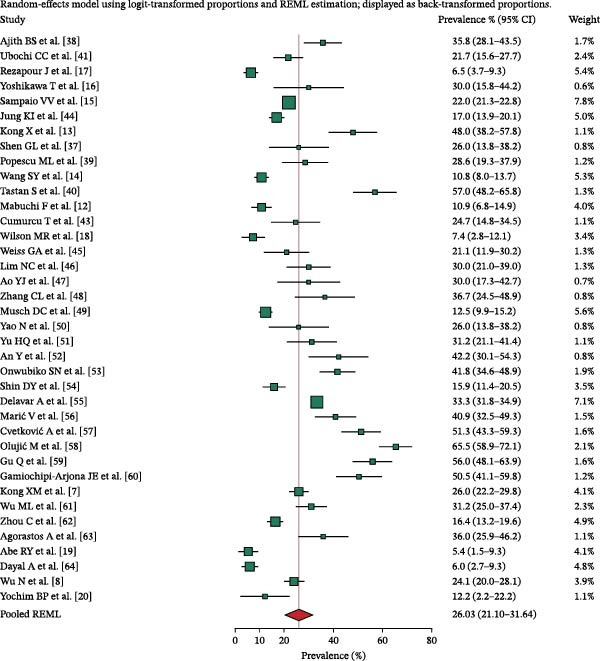
Forest plot of the pooled prevalence of depression among patients with glaucoma. Random‐effects model using logit‐transformed proportions and REML estimation. Square size is proportional to approximate random‐effects weight. Diamond indicates pooled back‐transformed prevalence; red vertical line indicates pooled prevalence.

Thirty‐eight studies, including 21,705 glaucoma patients and 5344 depression events, contributed to the prevalence analysis. The pooled prevalence of depression among glaucoma patients was 26.03% (95% CI, 21.10%–31.64%). Heterogeneity was substantial (*I* ^2^ = 95.95%; *τ* ^2^ = 0.6925; *p* < 0.001), and the prediction interval was broad (5.98%–66.04%). Study‐specific prevalence estimates are shown in Figure 3, and summary meta‐analytic findings are shown in Table 2.

### 3.7. Prevalence Sensitivity and Subgroup Analyses

The pooled prevalence remained stable after excluding self‐reported glaucoma studies (27.55%, 95% CI, 22.52%–33.18%), excluding the very large database study (26.14%, 95% CI, 21.07%–31.94%), and excluding the unclear‐ascertainment study (25.92%, 95% CI, 20.90%–31.67%). After excluding extreme prevalence estimates below 10% or above 50%, the pooled prevalence was 25.69% (95% CI, 21.99%–29.78%), heterogeneity decreased (*I* ^2^ = 93.91%), and the prediction interval narrowed to 10.47%–50.54%.

Subgroup analyses showed that the prevalence was 27.48% (95% CI, 22.36%–33.25%) in clinically or medically ascertained glaucoma studies and 8.63% (95% CI, 5.20%–14.01%) in self‐reported glaucoma studies. The prevalence was 17.10% (95% CI, 10.19%–27.26%) in population/database/community‐based studies and 25.32% (95% CI, 16.28%–37.16%) in hospital/clinic‐based studies. Differences by the detailed depression assessment method were statistically significant, but subgroup estimates were unstable where only one or two studies informed a category. See Supporting Information 7: Figure S3: Prevalence subgroup forest plot.

### 3.8. Longitudinal Narrative Synthesis

Longitudinal evidence was limited and could not be pooled. Zhang et al. [22], using UK Biobank data, suggested a longitudinal association between baseline glaucoma and incident hospitalized depression over a median 11.3 years of follow‐up (fully adjusted HR, 1.54; 95% CI, 1.01–2.34; *p* = 0.044). Lee et al. [23], a Korean nationwide cohort, reported a 1.13‐fold higher incidence of depression among newly diagnosed open‐angle glaucoma patients compared with matched non‐OAG controls, but the full HR and 95% CI for the glaucoma‐versus‐control comparison were not extractable; available HRs focused mainly on physical activity trajectories among OAG patients. Vidal et al. [21] reported no significant association between self‐reported glaucoma alone and incident depression in ELSA‐Brasil (relative risk ratio [RRR] = 1.38, 95% CI, 0.78–2.46; *p* = 0.26). Su et al. [65] did not compare glaucoma with non‐glaucoma controls but provided supportive evidence on factors associated with subsequent depression among glaucoma patients, including dementia, thyroid disease, headache, systemic beta‐blocker use, and calcium channel blocker use.

### 3.9. Certainty of Evidence (GRADE)

Using GRADE, the certainty of evidence was rated as very low for all three main outcome domains (Supporting Information 4: Supporting Tables S3 and S4). The comparative OR evidence was downgraded because it was observational, predominantly cross‐sectional or case‐control, based primarily on crude estimates, highly heterogeneous (*I* ^2^ = 95.95%), indirectly applicable across heterogeneous glaucoma populations and depression definitions, imprecise at the prediction interval level, and vulnerable to residual confounding and possible small‐study or design‐related bias. The single‐arm prevalence evidence was also rated as very low because of serious risk of bias, very serious inconsistency, indirectness related to different screening instruments and thresholds, and broad prediction intervals. Longitudinal evidence was rated as very low because only one directly extractable glaucoma‐versus‐control HR was available, and the remaining longitudinal studies differed in comparators, effect measures, and outcome definitions.

## 4. Discussion

### 4.1. Principal Findings

This systematic review and meta‐analysis found that glaucoma was associated with ~2.8‐fold higher odds of depression compared with non‐glaucoma controls. The finding persisted across multiple sensitivity analyses, including analyses excluding self‐reported glaucoma and the very large electronic health record study. In parallel, the pooled prevalence of depression among glaucoma patients was ~26%, although the prediction interval was wide, and individual study estimates varied markedly.

The GRADE assessment reinforces this cautious interpretation. Although the pooled estimates suggest a comparative association and a clinically meaningful occurrence of depressive outcomes among individuals with glaucoma, the certainty ratings across the comparative, prevalence, and longitudinal evidence domains support a conservative reading. Statistical significance in the pooled OR analysis should therefore not be interpreted as evidence of causality, temporality, or a precise effect magnitude across clinical settings.

The evidence should be interpreted as a noncausal association rather than evidence of a causal effect of glaucoma on depression. Most comparative studies were cross‐sectional or case‐control designs; therefore, they cannot establish temporality between glaucoma and depression. Important unmeasured or incompletely adjusted confounders include age, visual acuity, visual field severity, glaucoma duration, treatment burden, medication adverse effects, systemic comorbidities, socioeconomic stress, healthcare access, and pre‐existing mood disorders. Longitudinal evidence was suggestive but too sparse and methodologically heterogeneous for pooled estimation.

### 4.2. Comparison With Previous Literature

The overall direction is consistent with the included evidence summarized in Figures 2 and 3, Tables 1 and 2, and Supporting Information 4: Supporting Tables S2–S4. Compared with less differentiated syntheses, this review separately summarized comparative odds ratios, single‐group prevalence, and longitudinal evidence; used REML random‐effects models; reported prediction intervals; and conducted sensitivity analyses focused on major sources of clinical and methodological heterogeneity. The conservative primary estimate reflects analysis‐specific eligibility rules and avoidance of mixing prevalence, cross‐sectional odds ratios, and incident depression estimates in a single pooled result.

### 4.3. Possible Mechanisms

Several pathways may explain this association. Progressive visual field loss can reduce mobility, reading, driving, occupational function, and social participation [1–5]. Chronic medication use, ocular surface symptoms, cost of treatment, fear of blindness, and repeated clinical follow‐up may contribute to psychological distress [2–4, 6–8]. Biological pathways are also plausible, including shared genetic, vascular, inflammatory, neurodegenerative, sleep, circadian, or autonomic mechanisms, but the observational evidence in this review cannot establish the mechanism or directionality [22]. Depression may also worsen glaucoma care indirectly by reducing adherence and self‐management capacity [60, 70], and physical activity may be relevant to open‐angle glaucoma/depression trajectories [23].

### 4.4. Interpretation of Heterogeneity

Extreme heterogeneity was observed in both OR and prevalence analyses. Important contributors include differences in glaucoma ascertainment, depression measures and thresholds, recruitment setting, disease severity, population age, comparator type, and whether studies used clinical examinations, self‐report, or administrative codes. Self‐reported glaucoma studies showed weaker associations than clinically ascertained studies, supporting the view that exposure misclassification can dilute the effects. Conversely, hospital‐based studies may overrepresent more severe glaucoma, greater treatment burden, or higher comorbidity, inflating the depression prevalence relative to community samples. These patterns suggest that functional vision loss and its consequences may be more directly related to depressive outcomes than the diagnostic label of glaucoma alone. Depression measurement heterogeneity was a major source of inconsistency and indirectness: included studies used PHQ‐9, HADS‐D, SDS/Zung, GDS, CES‐D, BDI‐II, HAM‐D/HDRS, diagnostic codes, and structured interviews, with different thresholds for case definition. Lower screening thresholds may increase symptom‐positive prevalence, whereas diagnostic code definitions may identify more clinically severe or treated depression. These differences can shift both prevalence estimates and ORs and limit the interpretation of any single pooled estimate. Because cut‐off values were not reported consistently enough to define a defensible cross‐instrument extreme‐threshold category, we interpreted the sensitivity analysis excluding extreme prevalence estimates only as a pragmatic stability check, not as a scale‐specific threshold exclusion.

### 4.5. Clinical Implications

The findings support routine awareness of depression in glaucoma care, particularly among patients with advanced disease, visual impairment, poor quality of life, high treatment burden, or comorbid systemic illness. The results do not imply that every glaucoma patient requires psychiatric assessment, but they support practical pathways: brief symptom screening in high‐risk patients, attention to adherence and quality‐of‐life concerns, and referral to primary care or mental health services when clinically indicated. Integrated ophthalmology and mental health collaboration may improve patient‐centered care.

### 4.6. Strengths and Limitations

Strengths include an expanded multilingual search through March 2026, analysis‐specific extraction tables, separation of comparative, prevalence, and longitudinal evidence, quality assessment using NOS and JBI tools, REML random‐effects modeling, prediction intervals, subgroup analyses, multiple sensitivity analyses, and formal GRADE certainty‐of‐evidence assessment. Limitations include the predominance of cross‐sectional designs, very high heterogeneity, incomplete adjustment in pooled crude ORs, variation in depression instruments and glaucoma definitions, possible small‐study effects or design‐related asymmetry, and limited extractable longitudinal effect estimates. Formal interrater agreement was not calculated. Some single‐group prevalence studies required classification decisions where thresholds or diagnostic details were not fully harmonized; these decisions were handled through sensitivity analyses. One very large electronic health record study was available only as an ARVO Annual Meeting abstract without an identifiable DOI; therefore, its influence was evaluated in sensitivity analysis by excluding that study [15]. The GRADE assessment rated the certainty of evidence as very low for all main outcome domains [30], which should be considered when interpreting statistically significant pooled estimates.

We evaluated the extractability of glaucoma subtype information across the included studies. Some studies enrolled mixed glaucoma subtypes or clinically distinct entities such as acute angle‐closure glaucoma, pseudoexfoliative glaucoma, secondary glaucoma, ocular hypertension, or glaucoma suspects, while others did not report subtype‐specific depression or depressive‐symptom events. A reliable subtype‐stratified meta‐analysis was therefore not feasible. The pooled estimates should be interpreted as applying to heterogeneous glaucoma populations rather than to POAG or PACG specifically. Future studies should report glaucoma subtype, severity, visual field loss, visual acuity, treatment burden, and disease duration because these factors may be more directly related to depressive symptoms than the diagnostic label of glaucoma alone.

## 5. Conclusion

In this systematic review and meta‐analysis, individuals with glaucoma had a higher observed burden of depression or depressive symptoms compared with individuals without glaucoma, although the certainty of evidence was very low, and causal inference remains limited. Approximately one‐quarter of patients with glaucoma in included studies screened positive for depression or clinically relevant depressive symptoms. The observed association may reflect complex bidirectional relationships between glaucoma‐related visual burden and depressive symptoms. These findings justify greater clinical attention to mental health in glaucoma care, but they do not establish temporality or causality. Future studies should use standardized glaucoma definitions, validated depression instruments, severity‐stratified reporting, and adjusted longitudinal models that distinguish prevalent depressive symptoms from incident depressive disorders while accounting for visual severity, treatment burden, comorbidities, socioeconomic factors, and pre‐existing mood disorders.

## Author Contributions

Xuan Liao and Weihua Yang conceived and designed the study. Chenxuan Liu and Mengxiong Luo conducted the literature search, study screening, data extraction, and data verification. Xuan Liao and Chenxuan Liu performed the statistical analyses and interpreted the results. Xuan Liao and Chenxuan Liu drafted the manuscript. Mengxiong Luo and Weihua Yang critically revised the manuscript. Weihua Yang supervised the study.

## Funding

This work was supported by the Natural Science Foundation Key Project of Science & Technology Department of Sichuan Province (Grant 2026YFTX0072) and the Major Project on Quality Education and Teaching Reform in Sichuan Province (Grant YJGXM25‐A028).

## Disclosure

All authors reviewed and approved the final manuscript.

## Conflicts of Interest

The authors declare no conflicts of interest.

## Supporting Information

Additional supporting information can be found online in the Supporting Information section.

## Supporting information


**Supporting Information 1** PRISMA 2020 checklist (S02_PRISMA_2020_Checklist.docx). Provides the completed PRISMA 2020 checklist for the systematic review and meta‐analysis.

**Supporting Information 2** Search strategies and results (S01_Search_Strategies_and_Results.docx). Provides the full electronic search strategies and search results for PubMed, Embase, Cochrane Library, Web of Science, SinoMed, Wanfang, VIP, CNKI, ChiCTR, and ClinicalTrials.gov.

**Supporting Information 3** Data extraction file (S03_Data_Extraction_File.xlsx). Provides the study‐level extraction dataset used for the comparative odds‐ratio analyses, subgroup and sensitivity analyses, prevalence analyses, longitudinal evidence summary, quality assessment, and adjusted narrative synthesis.

**Supporting Information 4** Supporting tables and GRADE evidence profile (S04_Supporting_Tables_and_GRADE.xlsx). Provides Tables S1–S4, including detailed study characteristics, methodological quality assessments, subgroup and sensitivity analyses, longitudinal narrative synthesis, and GRADE summary/domain‐rationale tables.

**Supporting Information 5** Figure S1: Main odds‐ratio funnel plot (Suppl_Fig_S1_Main_OR_Funnel_Plot.pptx).

**Supporting Information 6** Figure S2: Trim‐and‐fill funnel plot (Suppl_Fig_S2_Trim_and_Fill_Funnel_Plot.pptx).

**Supporting Information 7** Figure S3: Prevalence subgroup forest plot (Suppl_Fig_S3_Prevalence_Subgroup_Forest_Plot.pptx).

## Data Availability

The data supporting the findings of this study are available in the article and supporting information. Further inquiries can be directed to the corresponding author.
