# Supplementary material for: Depression and Depressive Symptoms in Glaucoma: A Systematic Review and Meta‐Analysis of Comparative, Prevalence, and Longitudinal Evidence
Source: Depress Anxiety. 2026 Aug 14;2026:7344513. doi: 10.1155/da/7344513 (PMC13474487; doi:10.1155/da/7344513)
Supplement: Supplementary file 2 [file DA-2026-7344513-s002.docx]

**Total n=5663 2026.03.27**

**Pubmed n=613**

**Retrieval formula：(("Depression"[Mesh] OR "Depressive Disorder"[Mesh] OR depression[tiab] OR depressive[tiab] OR "depressive symptoms"[tiab])) AND ("Glaucoma"[Mesh] OR glaucoma[tiab] OR "primary open-angle glaucoma"[tiab] OR "primary angle-closure glaucoma"[tiab])**

**Embase n=756**

**Retrieval formula**

**(depression:ti,ab OR 'depressive symptoms':ti,ab) AND (glaucoma:ti,ab OR 'open angle glaucoma':ti,ab OR 'angle closure glaucoma':ti,ab)**

**Cochrane Library n=130**

**Retrieval formula**

**(depression:ti,ab,kw OR depressive:ti,ab,kw OR "depressive symptoms":ti,ab,kw) AND (glaucoma:ti,ab,kw)**

**Web of science n=692**

**Retrieval formula**

**TS = ((depression OR depressive OR "depressive symptoms") AND (glaucoma OR "primary open-angle glaucoma" OR "primary angle-closure glaucoma"))**

**Sinomed n=462**

**Retrieval formula：("抑郁"[摘要:智能] OR "抑郁症"[摘要:智能] OR "抑郁障碍"[摘要:智能] )AND ("青光眼"[摘要:智能])**

**Wanfang n=81**

**Retrieval formula**

**(标题或关键词:(抑郁症 OR 抑郁障碍) OR 主题:(抑郁症 OR 抑郁障碍)) AND (标题或关键词:(青光眼) OR 主题:(青光眼))**

**VIP n=99**

**Retrieval formula**

**M=(抑郁 OR 郁症 OR 抑郁障碍) AND M=青光眼**

**CNKI n=250**

**Retrieval formula：(SU='抑郁' OR SU='抑郁症' OR SU='抑郁障碍') AND SU='青光眼'**

**ChiCTR n=2543**

**Retrieval formula：抑郁 青光眼**

**ClinicalTrials.gov n=37**

**Retrieval formula：Condition/disease: glaucoma Other terms: depression**

**Condition/disease: glaucoma Other terms: depressive symptoms**
